# Analytically Sensitive *Rickettsia* Species Detection for Laboratory Diagnosis

**DOI:** 10.4269/ajtmh.21-0757

**Published:** 2022-03-14

**Authors:** Ida H. Chung, Lauren K. Robinson, Jeri J. Stewart-Juba, Gregory A. Dasch, Cecilia Y. Kato

**Affiliations:** Rickettsial Zoonoses Branch, Division of Vector-Borne Diseases, National Center for Emerging and Zoonotic Infectious Diseases, Centers for Disease Control and Prevention, Atlanta, Georgia

## Abstract

Clinical and laboratory diagnosis of rickettsial diseases is challenging because of the undifferentiated symptoms (commonly fever, headache, and malaise) and low bacteremia (< 100 genomic copies [gc]/mL) during the early acute stage of illness. Early treatment with doxycycline is critical for a positive outcome, especially in *Rickettsia rickettsii* (Rocky Mountain spotted fever) infections where cases may be fatal within 5 to 10 days from symptom onset, emphasizing the need for more sensitive diagnostics. A real-time reverse transcriptase polymerase chain reaction (PCR) assay, RCKr, was developed and validated for *Rickettsia* spp. nucleic acid detection in human clinical samples. The limit of detection for RCKr was determined to be 20 gc/mL, compared with our 2013 (Kato et al.) laboratory developed test, PanR8 at 1,800 to 2,000 gc/mL. Inclusivity, exclusivity, accuracy, and precision results correlated as expected. From an evaluation of 49 banked clinical samples, RCKr detected 35 previously positive samples, as well as two specimens that were PanR8 real-time PCR negative yet clinically diagnosed as possible rickettsiosis. Ct values from RCKr clinical sample testing show a 100-fold increase relative to PanR8. Additional testing is needed to understand the clinical sensitivity of RCKr; however, this study demonstrates RCKr to have high analytical specificity and sensitivity for *Rickettsia* detection.

## INTRODUCTION

*Rickettsia* species (spp.) are Gram-negative obligate intracellular *Alphaproteobacteria* and the causative agents of rickettsioses throughout the world. Classified according to their antigenic properties, two main groups are associated with human disease: spotted fever group *Rickettsia* (SFGR), responsible for diseases such as Rocky Mountain spotted fever (RMSF), African tick bite fever, and Mediterranean spotted fever; and typhus group *Rickettsia* which cause murine and epidemic typhus. The transitional group of *Rickettsia* is responsible for diseases such as rickettsialpox and flea-borne spotted fever. These rickettsioses often present with undifferentiated symptoms during early illness, such as fever, headache, and malaise, making clinical diagnosis difficult, so cases are often missed even in endemic areas or when severe.^1^^,^^10^^,^^15^ Certain SFGR present with rash or eschar (necrotic lesion), but these cannot be relied on for diagnosis. Epidemiologic information (e.g., travel, arthropod or animal exposure) and prompt clinical responses to doxycycline (the recommended treatment) also support clinical diagnosis. Early treatment is essential to increase the probability of a positive prognosis, especially in RMSF, for which a delay in doxycycline administration is correlated with more severe outcomes and death.^10^

The serological standard for confirmation of rickettsioses is the observation of a ≥ 4-fold rise in patient IgG antibody titers by indirect immunofluorescence antibody assay (IFA) to rickettsial antigens in paired acute (first 2 weeks of illness or while symptomatic) and convalescent samples taken 2 to 4 weeks later.^3^^,^^15^ Recently, molecular detection by real-time polymerase chain reaction (PCR) of rickettsial DNA in clinical specimens has become more widely used for accurate specific confirmation of rickettsial infections in patients.^8^^,^^9^ Although early treatment is beneficial, samples must be obtained before or within 48 hours of treatment or PCR sensitivity is reduced. Although eschar swabs and tissue samples may contain abundant organisms, rickettsial bacteremia is generally observed to be low, at < 100 genomic copies (gc)/mL in peripheral blood, except in advanced disease.^6^^,^^7^^,^^12^ Even at the lower limits of detection (LoD) for real-time PCR, an efficiently repeatable assay at ∼9 DNA gc/reaction (5 µL of sample) still requires ∼1,800 gc of organism in 1 mL of blood.^6^ These factors are particularly troubling in the case of RMSF because of the rapid progression of this illness. While next generation sequence analyses of metagenomic samples are becoming more commonplace, most molecular assays target DNA of single copy genes in the rickettsial genome.^8^ We describe a real-time reverse transcriptase PCR (rtRT-PCR) assay, RCKr, for the detection of *Rickettsia* spp. including those known to be pathogenic to humans.

## MATERIALS AND METHODS

The RCKr assay (PanR6 in U.S. Patent Application 2021/0079453) was developed to detect *Rickettsia* spp. targeting both 23S rDNA and rRNA in total nucleic acid (TNA) extractions. Primers and probe were designed using the Integrated DNA Technology OligoAnalyzer Tool (IDT; https://www.idtdna.com/pages/tools/oligoanalyzer). The rtRT-PCR assay was performed using 5 µL TNA and qScript XLT 1-Step RT-qPCR ToughMix, Low-ROX (Quantabio, Beverly, MA) with the RCKr primer/probe set (forward primer: GGTCCCACAGACTTACCAAACTCA, reverse primer: TCGACTATGGACCTTAGCACCCAT, and probe labeled with a fluorescein (Fl) and black hole quencher-1 (BHQ1): Fl-CCGAATGTCGATGAGTACAGCATAGCAGAC-BHQ1). The Applied Biosystems 7500 Fast Dx Real-time PCR Instrument (Thermo Fisher Scientific, Waltham, MA) was used for testing with the following cycling parameters: 50°C for 10 min, 95°C for 1 min, and 45 cycles of 95°C for 10 sec, and 60°C for 1 min. Ramp rates for all steps were set at 7500 fast conditions, and fluorescence data was acquired at the end of each annealing step. PanR8, an existing real-time PCR assay developed for *Rickettsia* species DNA detection, was used for comparison with the RCKr assay. PanR8 primer and probe sequences and testing parameters were used according to Kato et al.^7^

*R. rickettsii* AZ3 strain isolated and cultured at the Rickettsial Zoonoses Branch, Centers for Disease Control and Prevention (Atlanta, GA), was used to assess assay performance.^13^^,^^14^ *R. rickettsii* organism from cell culture supernatant was suspended in sucrose phosphate glutamine (SPG) buffer and quantified using the PanR8 assay. EDTA whole blood was collected from a healthy donor (institutional review board [IRB] protocol number IRB00045947) through the Emory University Centers for Transfusion and Cellular Therapies (Atlanta, GA). The *R. rickettsii* suspensions were spiked into blood at 10-fold serial dilutions from 20,000 to 2 gc/mL. TNA was extracted using the MagNA Pure Compact Instrument and the MagNA Pure Compact Nucleic Acid Isolation Kit I (Roche Diagnostics, Indianapolis, IN) with an external lysis protocol. The extractions were performed with 280 µL of the MagNA Pure LC Lysis/Binding Buffer (Roche Diagnostics, Indianapolis, IN) and 20 µL of proteinase K (Sigma-Aldrich Inc., St. Louis, MO) added to 200 µL of the contrived blood sample, then incubated for 30 minutes at room temperature and eluted in 200 µL. The extracted TNA was evaluated using RCKr and PanR8 for reproducibility testing with nine total replicates.

Inclusivity for RCKr was assessed using nine *Rickettsia* species TNA extracted from culture grown organisms (Table 1). Exclusivity was evaluated for the RCKr assay by testing seven *Rickettsia* near neighbors, seven human tissues, nine environmental background bacteria, ten bacteria that cause diseases with similar symptoms, and two *Proteus* species that exhibit antigenic cross-reactivity with *Rickettsia* (Table 1). For accuracy, 30 TNA extracts from contrived blood specimens containing concentrations of *R. rickettsii* at 1,000, 100, 10, and 1 copy per reaction were evaluated. Ten additional blood specimens containing no organism were included as negative samples. The specimens were randomized and blinded by one operator, then extracted for TNA and tested by a second operator. Intra-assay reproducibility testing was performed using TNA extracted from 10 contrived blood specimens containing variable concentrations (1,000, 100, 10, and 1 copy per reaction) of *R. rickettsii* on the same run, in triplicate. Interassay reproducibility testing was performed using 1 TNA at 1 copy per reaction assessed over the course of 10 runs with multiple operators. To validate the use of the RCKr assay on diagnostic samples (IRB protocol number IRB7014), 49 banked clinical samples from 35 fatal and nonfatal cases of suspected rickettsioses were tested using RCKr and PanR8 assays (Table 2).

**Table 1:** Analytical evaluation of the Pan-*Rickettsia* real-time reverse transcriptase polymerase chain reaction assay, RCKr, for specificity (inclusivity and exclusivity), accuracy, and precision

Group	Samples/organisms	Result	CV*
Inclusivity	*Rickettsia* species, *n* = 9 (*R. rickettsii, R. parkeri, R. conorii, R. akari, R. amblyommatis, R. felis, R. prowazekii, R. typhi, R. africae*)	Positive	0.09–3.74%
Exclusivity	Human tissue, *n* = 7 (heart, kidney, liver, lung, peripheral blood leukocyte, spleen, skin)	Negative	N/A
	*Rickettsia* near neighbors, *n* = 7 (*Anaplasma phagocytophilum, Bartonella henselae, Bartonella quintana, Ehrlichia chaffeensis, Orientia tsutsugamushi* Gilliam, *O. tsutsugamushi* Karp, *O. tsutsugamushi* Kato)	Negative	N/A
	Environmental background bacteria, *n* = 9 (*Escherichia coli*, *Propionibacterium acnes*, *Enterococcus faecalis*, *Mycobacterium tuberculosis*, *Staphylococcus aureus* serotype 3, *Staphylococcus aureus* Seattle 1945, *Staphylococcus epidermidis* PCI 1200, *Staphylococcus epidermidis* Evans, *Streptococcus pyogenes*)	Negative	N/A
	Bacteria that cause diseases with similar symptoms, *n* = 10 (*Borrelia burgdorferi*, *Coxiella burnetii, Legionella pneumophila*, *Leptospira interrogans*, *Listeria monocytogenes*, *Neisseria gonorrhoeae*, *Neisseria meningitidis*, *Salmonella enterica* MZ1445, *Salmonella enterica* Typhi, *Salmonella enterica* Typhimurium)	Negative	N/A
	Cross-reactive *Proteus* species, *n* = 2 (*Proteus vulgaris, Proteus mirabilis*)	Negative	N/A
Accuracy	Blind panel of positive samples (30 contrived samples at four concentrations of 1000, 100, 10, and one copy per reaction)	Positive	1.38–2.39%
	Blind panel of negative samples (10 contrived samples with no organism)	Negative	N/A
Precision	Intra-assay reproducibility (10 contrived samples at 4 concentrations of 1000, 100, 10, and 1 copy tested in the same run, in triplicate)	Positive	0.40–1.60%
	Interassay reproducibility (one contrived sample at one copy per reaction tested over the course of 10 runs with multiple testers)	Positive	1.97%

* CV = Coefficient of variation, which is calculated using the following equation for each species and each concentration: [(standard deviation) / average] × 100%. Ranges show the lowest and highest variation within the panel.

**Table 2:** Sample type, patient outcome, and PCR results for 49 banked patient samples tested for *Rickettsia*

Sample no.	Patient no.	Sample type	Patient outcome	Days from onset	Days on DC	Patient rickettsial diagnosis*	PanR8 DNA detection† (Ct average)	RCKr rRNA and rDNA detection† (Ct average)	Species identification§
1	1	Serum¶	Fatal	7	3	RMSF	26.16	18.75	Rri positive
2‖	1	Blood¶	Fatal	7	3	RMSF	28.77	19.37	Rri positive
3‖	2	Blood¶	Fatal	5	3	RMSF	28.43	22.52	Rri positive
4	2	Spleen¶	Fatal	5	3	RMSF	25.53	18.37	Rri positive
5	2	Kidney¶	Fatal	5	3	RMSF	27.76	20.11	Rri positive
6	2	Lung¶	Fatal	5	3	RMSF	26.37	19.13	Rri positive
7	2	Liver¶	Fatal	5	3	RMSF	29.11	22.58	Rri positive
8	2	Skin¶	Fatal	5	3	RMSF	28.65	23.14	Rri positive
9	3	Brain¶	Fatal	9	Unk	RMSF	33.39	26.04	Rri positive
10	4	CSF	Fatal	9	Unk	RMSF	35.52	34.84	Rri positive
11	5	CSF	Fatal	5	3	RMSF	30.45	24.59	Rri positive
12	5	Serum	Fatal	5	3	RMSF	30.30	25.50	Rri positive
13	6	Serum	Fatal	7	3	RMSF	26.28	19.08	Rri positive
14	7	Plasma	Fatal	2	1	RMSF	30.35	28.70	Rri positive
15	7	Serum	Fatal	2	1	RMSF	28.71	22.03	Rri positive
16	7	CSF	Fatal	2	1	RMSF	26.45	21.75	Rri positive
17	8	Plasma	Fatal	2	Unk	RMSF	26.95	25.52	Rri positive
18	8	Serum	Fatal	2	Unk	RMSF	26.40	20.11	Rri positive
19*∥*	9	Blood CSF¶	Nonfatal	2	Unk	RMSF	37.39	31.66	Rri negative
20	10	Blood¶	Nonfatal	11	1	RMSF	37.23	34.10	Rri positive
21	11	Blood¶	Nonfatal	2	Unk	RMSF	37.85	32.40	Rri positive
22*∥*	11	Blood¶	Nonfatal	1	Unk	RMSF	32.34	26.23	Rri positive
23*∥*	12	Blood¶	Nonfatal	Unk	Unk	Epidemic typhus	30.67	26.30	Rpr positive
24*∥*	13	Blood¶	Nonfatal	6	0	Epidemic typhus	34.73	31.07	Rpr positive
25	14	Blood¶	Nonfatal	11	0	Rickettsiosis	38.63	32.78	Rpr negative
26	15	Blood	Nonfatal	11	Unk	Murine typhus	37.78	33.84	Rty positive
27	16	Plasma	Nonfatal	Unk	Unk	RMSF	37.51	37.71	Rri positive
28	17	Bone marrow	Nonfatal	6	0	Murine typhus	30.43	31.00	Rty positive
29	18	Swab	Nonfatal	26	Unk	Rickettsiosis	32.98	32.03	ND
30	19	Swab	Nonfatal	15	4	Rickettsiosis	33.34	30.47	Rri negative
31	19	Eschar	Nonfatal	15	4	Rickettsiosis	26.21	27.51	Rri negative
32	19	Blood	Nonfatal	8	0	Rickettsiosis	Negative‡	37.42#	ND
33	20	Blood¶	Nonfatal	10	0	Not Rickettsiosis	Negative‡	Negative‡	Rpr negative
34	21	Blood¶	Nonfatal	5	3	Not rickettsiosis	Negative‡	Negative‡	Rri negative
35	22	Blood¶	Nonfatal	4	0	Not rickettsiosis	Negative‡	Negative‡	Rri negative
36	23	Blood¶	Nonfatal	2	Unk	Not rickettsiosis	Negative‡	Negative‡	Rri negative
37	24	Blood¶	Nonfatal	15	10	Not rickettsiosis	Negative‡	Negative‡	Rri negative
38	25	Blood¶	Nonfatal	19	2	Ehrlichiosis	Negative‡	Negative‡	Ech positive
39	26	Blood¶	Nonfatal	Unk	Unk	Anaplasmosis	Negative‡	Negative‡	Aph positive
40	27	Blood	Nonfatal	4	1	Not rickettsiosis	Negative‡	Negative‡	ND
41	28	Serum	Nonfatal	9	5	Not rickettsiosis	Negative‡	Negative‡	ND
42	29	Serum	Unk	5	Unk	RMSF	34.78	32.96	Rri positive
43	30	Swab	Unk	7	1	Rickettsiosis	35.32	31.12	ND
44	30	Swab	Unk	8	2	Rickettsiosis	33.50	30.17	ND
45	31	Serum	Unk	11	0	Rickettsiosis	36.48	36.28	Rri negative
46	32	Serum	Unk	7	2	Rickettsiosis	Negative‡	39.23#	ND
47	33	Serum	Unk	10	0	Not rickettsiosis	Negative‡	Negative‡	ND
48	34	Serum	Unk	8	0	Not rickettsiosis	Negative‡	Negative‡	ND
49	35	Serum	Unk	7	1	Not rickettsiosis	Negative‡	Negative‡	ND

Aph = *Anaplasma phagocytophilum*; CSF = cerebrospinal fluid; DC = doxycycline; Ech = *Ehrlichia chaffeensis*; ND = Not determined; PCR = polymerase chain reaction; RMSF = Rocky Mountain spotted fever; Rri = *Rickettsia rickettsii*; Rpr = *R. prowazekii*; Rty = *R. typhi*; Unk = unknown (data unavailable).

* Patient diagnosis based on compatible clinical signs and epidemiologic data and confirmed by real-time PCR or sequencing; Rickettsiosis refers to an undetermined rickettsial species; RMSF, per detection of *R. rickettsii* DNA by real-time PCR.

† Using the slope of a 5-point, 10-fold dilution series of quantified *Rickettsia* nucleic acid, reaction efficiency was calculated for the RCKr (104.3%) and PanR8 (95.9%) assays. The RCKr assay gene target is 23S rRNA. The PanR8 assay gene target was mislabeled in Kato et al.^7^ and should be the 50S ribosomal protein L16.

‡ Negative = no detectable *Rickettsia* nucleic acid in sample.

§ Additional species identification performed by real-time PCR or nested PCR, followed by sequencing.

‖ Positive specimens used to evaluate increases in RCKr assay target detection. Ten-fold serial dilutions were detected at increases from 100 to 1,000,000-fold: sample 19 at 100-fold, sample 24 at 1,000-fold, samples 22 and 23 at 10,000-fold, and samples 2 and 3 at 1,000,000-fold.

¶ A new aliquot (200 µL, serum/blood) or segment (tissue) of the original specimen (aliquoted or portioned upon receipt and stored at −80°C) was extracted for TNA before testing with the PanR8 and RCKr assays.

# PCR products from samples tested positive by the RCKr assay alone was verified for correct band size (91 bp) using gel electrophoresis.

## RESULTS AND DISCUSSION

**Figure 1.**
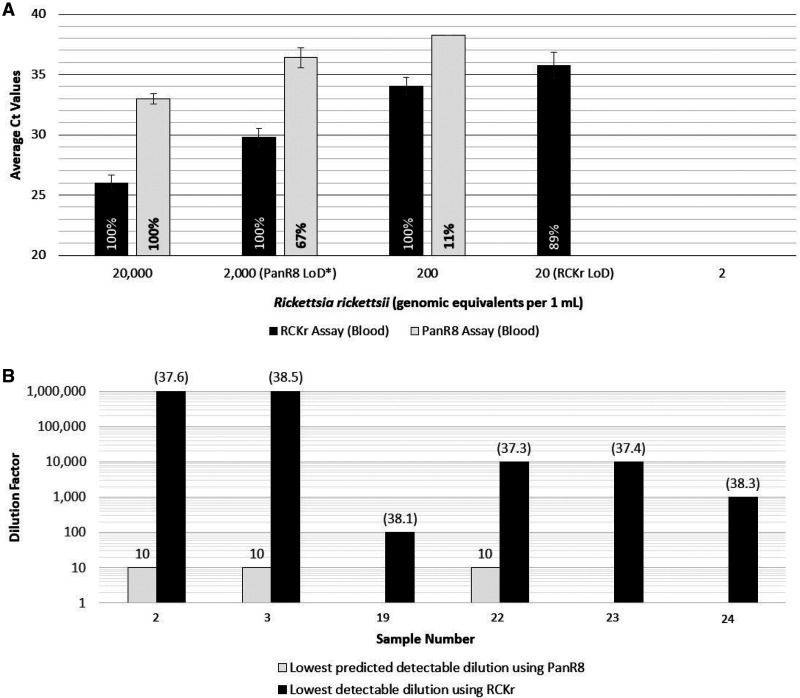
(**A**) Limit of detection (LoD) assessment using contrived samples of Rickettsia rickettsii organism spiked into blood at 10-fold dilutions of 20,000 to two genomic equivalents per 1 mL. Three sets of blood samples at each concentration were extracted for TNA. All extracts were tested with the RCKr (rtRT-PCR) and PanR8 (rtPCR) assays for a total nine replicates. The number of positive replicates for each data set is represented as a percentage (reproducibility). *Expected PanR8 assay LoD7. (**B**) Detectable limits of Rickettsia in acute blood of patients using the PanR8 and RCKr assays. The lowest detectable dilution by DNA detection was estimated based on quantification values and the dilution meeting parameters of the established PanR8 assay LoD at nine copies/5 µL. The Ct values are in parentheses for the last dilution where Rickettsia was detectable.

Extracted TNA from contrived blood samples spiked with *R. rickettsii* at 10-fold serial dilutions from 20,000 to 2 gc/mL were tested to assess analytical sensitivity (Figure 1A). The LoD for RCKr was determined to be 20 gc/mL at 89% reproducibility (8/9 replicates). Originally described with an LoD of 1,800 gc/mL, PanR8, in this dilution series for comparison, detected 2,000 gc/mL with 67% reproducibility, representing a ∼100-fold increase in detection for RCKr. Inclusivity tests using TNA from culture grown organisms from nine *Rickettsia* species were positive with RCKr, whereas exclusivity assessment consisting of 35 TNAs tested negative (Table 1). High accuracy and precision were determined using contrived blood specimens, and results correlated as expected even with equivocal PanR8 samples at one copy per reaction (Table 1).

Forty-nine banked clinical samples were assessed using both RCKr and PanR8. In 18 clinical samples (1–18, Table 2) from eight fatal RMSF cases which included blood, serum, cerebrospinal fluid, plasma, brain, spleen, kidney, lung, liver, and skin, RCKr detected rickettsial target sequences an average of 5.7 (range 0.54 to 9.41) Ct values lower than PanR8, whereas in 13 samples (19–31) from 11 nonfatal patients that included blood, plasma, bone marrow, swab, and eschar samples, RCKr detected rickettsial target nucleic acid at an average of 3.1 (range –1.3 to 6.12) Ct values lower than PanR8. Ct values of three specimens (27–29) were detected at similar values for both assays, and one specimen (31) had a Ct value difference of 1.3 higher with RCKr than PanR8 (Table 2). This could be due to nucleic acid degradation. Samples from patient 19 included swab, eschar, and blood (samples 30–32, Table 2). The blood, sample 32, collected on day 8 post–symptom onset (pso) and before doxycycline administration, was negative when originally tested with PanR8, and positive with RCKr. The negative PanR8 and high RCKr Ct values indicate undetectable or very low rickettsial copy numbers. The swab and eschar samples (30–31) from patient 19, collected 15 days pso and 4 days post–doxycycline treatment, were positive by PanR8 and RCKr.

Of the eight specimens (42–49, Table 2) collected from seven patients with unconfirmed rickettsioses, four specimens (three patients) were positive by PanR8 and RCKr, and one specimen (one patient) was positive by RCKr alone (amplicon size verified using gel electrophoresis). It remains unclear whether the RCKr positive and PanR8 negative patient sample represents an increase in *Rickettsia* detection or a false positive; although diagnosed clinically as rickettsiosis and with amplicon size verification, species identification through real-time PCR or sequencing attempts were unsuccessful (possibly due to low DNA quantities). Twelve specimens previously determined to be negative by PanR8 (33–41 and 47–49, Table 2) were also negative by RCKr. Of these 12, 10 etiologies remain undefined, whereas samples 38 and 39 previously tested positive by PCR for *Ehrlichia chaffeensis* and *Anaplasma phagocytophilum*, respectively. RCKr detected all previously defined positive samples as well as two additional specimens that PanR8 was not able to detect.

When comparing the performance of PanR8 and RCKr, lower Ct values indicate a higher number of detectable PCR target sequences and therefore the potential for better detection. The estimated increases in RCKr detectable targets were observed from 10-fold serial dilutions of *Rickettsia* positive sample extracts (2, 3, 19, 22-24, Table 2), which shows detection at 100 to 1,000,000-fold dilutions (Figure 1B). The variability in the difference between DNA and TNA detection probably reflects the presence and stability of rRNA in individual samples. It is unknown how this difference is affected by disease severity, day collected, sample age, or doxycycline treatment.

Because IFA is the reference standard for rickettsial disease confirmation, it would be valuable to assess acute samples collected from patients who demonstrated seroconversion (4-fold change in IFA titer) to better assess the performance of these assays. Although swabs, eschars, and tissue samples contain abundant rickettsial organisms during acute illness, blood and serum typically contain lower quantities due to low levels of circulating bacteria, which may not be measurable with PanR8.^16^ However, RCKr could provide more reproducible detection because of the lower Ct values and higher abundance of rRNA targets within a sample. This improvement may provide further insight into the early stages of disease in these sample types.

23S rRNA is constitutively expressed and bound by ribosomal proteins and is therefore potentially more stable and in higher copy number than the more traditionally designed DNA or mRNA targets. More 23S rRNA copies per *Rickettsia* cell are present than the single copy chromosomal DNA target. The 23S ribosomal RNA target region is in the operon that includes the methionyl-tRNA_f_^Met^ formyltransferase (*fmt*), 23S rRNA, and 5S rRNA gene cluster, which are well conserved among *Rickettsia* including those that are pathogenic to humans. This gene arrangement is unlike the 16S (rrs), 23S, and 5S rRNA operon observed in many other bacteria. This uncommon arrangement seen in *Rickettsia* is thought to have occurred before *Rickettsia* spp. and *Orientia* spp. diverged.^2^ This gene arrangement, and more specifically, the conserved sequences flanking the 23S rRNA and 5S rRNA intergenic region has previously served as a target for *Rickettsia* detection, whereas sequence variation in the intergenic region is used for differentiation of species.^5^ The 16S rRNA gene has also been used for *Rickettsia* detection because of the highly conserved sequences found among the species in this genus.^4^^,^^11^

Relatively little is documented about the stability of ribosomes and rRNA in *Rickettsia* under different storage conditions. Although samples drawn after 48 hours of doxycycline administration may have reduced rickettsial content, we were able to detect rickettsial DNA and rRNA in specimens drawn after 72 hours (5–7 days pso) in patients with fatal outcomes. Swabs and eschar samples from nonfatal rickettsiosis patients remained positive at 15 or more days pso. The swab and eschar (patient 19) collected 15 days pso were sampled 96 hours post doxycycline. These results show detectable *Rickettsia* may be present in samples such as swab and eschar outside of the recommended collection timeframe (e.g., within 14 days pso and before or within 48 hours of doxycycline treatment), adding to the significance of collecting specimens while the patient is symptomatic and not restricting sample collection to a general timeframe of 14 days.

## CONCLUSION

We show an increase in detectable nucleic acid by 100-fold in clinical specimens with more reproducible Ct values when including 23S rRNA as a target, therefore improving the accuracy of rickettsial detection. Future work includes evaluation of additional samples throughout the course of infection to determine the utility of this protocol in other rickettsial species of interest and to better understand assay limitations for agent detection at the early stage of illness. Regardless, our results represent an important improvement in effective laboratory diagnosis of rickettsioses.
